# Fructose-1,6-bisphosphate aldolase encoded by a core gene of *Mycoplasma hyopneumoniae* contributes to host cell adhesion

**DOI:** 10.1186/s13567-018-0610-2

**Published:** 2018-11-19

**Authors:** Yanfei Yu, Maojun Liu, Lizhong Hua, Mingjun Qiu, Wei Zhang, Yanna Wei, Yuan Gan, Zhixin Feng, Guoqing Shao, Qiyan Xiong

**Affiliations:** 1 Key Laboratory of Veterinary Biological Engineering and Technology of Ministry of Agriculture, Institute of Veterinary Medicine, Jiangsu Academy of Agricultural Sciences, Nanjing, China; 2 Key Lab of Food Quality and Safety of Jiangsu Province-State Key Laboratory Breeding Base, Nanjing, China; 3 College of Animal Science and Technology, Shanxi Agricultural University, Taigu, China; 4 Key Lab of Animal Bacteriology of Ministry of Agriculture, College of Veterinary Medicine, Nanjing Agricultural University, Nanjing, China

## Abstract

*Mycoplasma hyopneumoniae* is an important respiratory pathogen that causes great economic losses to the pig industry worldwide. Although some putative virulence factors have been reported, pathogenesis remains poorly understood. Herein, we evaluated the relative abundance of proteins in virulent 168 (F107) and attenuated 168L (F380) *M. hyopneumoniae* strains to identify virulence-associated factors by two-dimensional electrophoresis (2-DE). Seven proteins were found to be ≥ 1.5-fold more abundant in 168, and protein–protein interaction network analysis revealed that all seven interact with putative virulence factors. Unexpectedly, six of these virulence-associated proteins are encoded by core rather than accessory genomic elements. The most differentially abundant of the seven, fructose-1,6-bisphosphate aldolase (FBA), was successfully cloned, expressed and purified. Flow cytometry demonstrated the surface localisation of FBA, recombinant FBA (rFBA) mediated adhesion to swine tracheal epithelial cells (STEC), and anti-rFBA sera decreased adherence to STEC. Surface plasmon resonance showed that rFBA bound to fibronectin with a moderately strong *K*_D_ of 469 nM. The results demonstrate that core gene expression contributes to adhesion and virulence in *M. hyopneumoniae*, and FBA moonlights as an important adhesin, mediating binding to host cells via fibronectin.

**Electronic supplementary material:**

The online version of this article (10.1186/s13567-018-0610-2) contains supplementary material, which is available to authorized users.

## Introduction

*Mycoplasma hyopneumoniae* is the etiological agent of porcine enzootic pneumonia, one of the most damaging respiratory diseases affecting pig farming. Despite low direct mortality, *M. hyopneumoniae* can lower the feed conversion efficiency and reduce the growth rate, resulting in large economic losses.

The *M. hyopneumoniae* pathogen colonises and destroys the epithelial surfaces of the respiratory tract [1], and adhesion to the respiratory epithelium is the first and most important step in infection [2, 3]. Several proteins are involved in adhesion, including P97, the first adhesin to be identified in this species, which binds to the cilia of respiratory epithelial cells via its C-terminal R1 domain [4]. Other adhesion factors, such as P102 [5, 6], P159 [7], P146 [8], P216 [9], P116 [10], Mhp271 [11], Mhp683 [12], Mhp107 [13] and so on have since been reported. Not only these adhesins, but also multifunctional cytosolic proteins “moonlighting” at the cell surface contribute to *M. hyopneumoniae* adhesion [14]. They include the following: MHJ_0125, a glutamyl aminopeptidase that moonlights as an adhesin on the surface of *M. hyopneumoniae* [15]; MHJ_0461, a leucine aminopeptidase which binds to heparin, plasminogen and foreign DNA and functions as an accessory adhesin [16]; and l-lactate dehydrogenase, an immunogenic cytoplasmic protein involved in the glycolytic process but also present at the cell surface with adhesin functions [17]. Glyceraldehyde 3-phosphate dehydrogenase (MHJ_0031) has also been identified as a putative moonlighting protein because it was predicted within a putative heparin-binding region [18]. Finally, elongation factor thermo unstable (EF-Tu), functions as an adhesin on the surface of *M. hyopneumoniae* by binding to multiple host proteins [19, 20]. However, despite these findings, the exact mechanisms responsible for pathogenesis and potential virulence factors in *M. hyopneumoniae* remain poorly understood [21].

Although infection by *M. hyopneumoniae* is highly prevalent (ranging between 38 and 100%) in almost all areas of pig production worldwide, there are discrepancies in terms of pathogenicity among isolates of *M. hyopneumoniae*. Comparative proteomics research, which offers a systematic analysis, can reveal novel putative virulence factors between bacterial strains in which virulence differs. For example, the available proteome map of *M. hyopneumoniae* strain 7448 served as a reference for comparative analysis of differentially virulent *M. hyopneumoniae* strains [22]. A subsequent proteomic comparison of *M. hyopneumoniae* pathogenic strain 232 and avirulent strain J revealed 11 differentially abundant proteins [23]. However, differences are evident among the genome sequences of *M. hyopneumoniae* strains. For example, the size of the genome ranges from 892 758 bp (strain 232, AE017332) to 964 503 bp (strain KM014, CP022714). Furthermore, differences are magnified after translation into proteins, due to variation in regulation and modification at both RNA and protein levels. Thus, the results obtained from comparative proteomics analyses of different sources of bacterial strains can be confusing, preventing the ability to focus on the most critical and common factors [24]. Subjecting strains with a consistent genomic background but differences in virulence to comparative proteomics analysis is one potential solution. To this end, we obtained the attenuated *M. hyopneumoniae* strain 168L (F380) from continuous passage of pathogenic strain 168 (F107) in cell-free medium [25]. Herein, we performed comparative proteomic analysis on these strains and identified a number of putative virulence-associated proteins. We demonstrate how pan-genome dynamics, linked to conserved and transposable elements, may help in the characterisation of virulence factors identified via comparative proteomics analysis. The findings highlight novel virulence-associated factors and the biological versatility of known proteins, leading to a more complete understanding of the complex physiological and infectious processes operating in *M. hyopneumoniae*.

## Materials and methods

### Ethics statement

All animal experiments were approved by the Committee on the Ethics of Animal Experiments and performed in Jiangsu Academy of Agricultural Sciences (License No. SYXK (Su) 2015-0019). The experimental procedures conformed to the guidelines of Animal Regulations of the Jiangsu Province (Government Decree No. 45) in accordance with international law.

### Bacterial strains and growth conditions

*Mycoplasma hyopneumoniae* strain 168 was isolated in Gansu Province, China, from a pig exhibiting typical characteristics of mycoplasmal pneumonia of swine (MPS) [26]. This field strain *was* cultured in KM2 cell-free liquid medium (a modified Friis medium) containing 20% (v/v) swine serum at 37 °C, and was gradually attenuated by continuous passage to the 380^th^ passage, yielding strain168L [25].

### Evaluation of virulence in *M. hyopneumoniae* strains 168 and 168L

Nine non-immunised cross-bred (Xiaomeishan × Landrace) 50-day-old snatch-farrowed, porcine-colostrum-deprived (SF-pCD) piglets were raised according to the methods described by Huang et al. [27, 28]. All nine piglets used to evaluate the virulence of *M. hyopneumoniae* strains 168 and 168L were free of sera IgG antibody recognising classical swine fever virus (CSFV Antibody Test Kit, IDDEXX Laboratories, USA), porcine reproductive and respiratory syndrome virus (PRRSV Antibody Test Kit, IDEXX Laboratories, USA), porcine pseudorabies virus (PRV Antibody Test Kit, IDEXX Laboratories, USA), porcine circovirus type 2 (PCV2 Antibody Test Kit, JBT, South Korea), and *M. hyopneumoniae* (*M. hyopneumoniae* Antibody Test Kit, IDEXX Laboratories, USA). They were also free of secretory IgA antibody recognising *M. hyopneumonia*e [29], and antigen of PCV2 [30] and *M. hyopneumoniae* [29]. The piglets were divided randomly into three groups (three piglets/group) and raised in three separate rooms. The piglets were intratracheally challenged with 5 mL of a 10^8^ colour change unit (CCU)/mL culture of strains 168 or 168L (or KM2 medium as a negative control). The piglets were observed daily for clinical signs of pneumonia, such as coughing and asthma. All animals were euthanised at 28 days after challenge, and lung lesions were scored using the rule of 28 method [31]. A value was assigned to seven pulmonary lobes based on the average extent of mycoplasmal lesions. Each pulmonary lobe was scored for severity of mycoplasmal lesions ranging from 0 to 4 (0 = absence of lobular pneumonia; 1 = 1–25% lesions; 2 = 26–50% lesions; 3 = 51–75% lesions; 4 = 76–100% lesions). For each animal, the sum of seven pulmonary lobes ranged from 0 to 28. All data were analysed by analysis of variance (ANOVA) using SPSS 20.0 software, and differences were considered significant at *p* ≤ 0.05.

### Protein extraction and two-dimensional electrophoresis (2-DE)

Cultures of strains 168 and 168L were harvested by centrifugation at 16 000 × *g* for 20 min at 4 °C at the late exponential phase of growth. Pellets were washed three times with 10 mM TRIS–HCl (pH 7.4) and resuspended in protein extract consisting of 1.52 g thiourea, 4.2 g urea, 0.4 g CHAPS, 200 µL amphoteric electrolyte, 61.6 mg dithiothreitol (DTT; all Bio-Rad), and protease inhibitor (Merck) dissolved in 10 mL ultrapure water. After vortexing and centrifugation, total protein in the supernatant was subjected to cleanup with a ReadyPrep 2-D cleanup kit (Bio-Rad). Purified proteins were redissolved in 350 µL rehydration solution (7 M urea, 2 M thiourea, 0.001% bromophenol blue; Bio-Rad) and centrifuged to remove insoluble components. Samples were finally loaded onto a 17 cm strip (pH 3–10; Bio-Rad) and isoelectric focusing (IEF) was carried out at 20 °C by positively rehydrating at 50 V for 12 h, increasing slowly to 250 V for 1 h, rapidly to 1000 V for 1 h, 10 000 V for 3 h, rapidly to 10 000 V to a total of 90 000 Vh, then rapidly to 500 V. Sodium dodecyl sulphate-polyacrylamide gel electrophoresis (SDS-PAGE) was then performed according to a previous protocol [24].

### Image analysis, MALDI-TOF–MS/MALDI-TOF-TOF–MS, and database searching

After Coomassie blue staining of the SDS-PAGE gel, the detected protein spots from each gel were matched automatically using PDQuest V8.0 software with additional visual analysis. The intensity of individual spot was normalised relative to the total valid spot intensity for each gel. Protein expression levels were calculated as the fold-change. Only spots for which the abundance in strain 168 was ≥ 1.5-fold higher than in strain 168L (Student’s *t* test ≥ 0.05) were excised from the 2-DE gel and subjected to matrix-assisted laser desorption/ionisation time-of-flight mass spectrometry (MALDI-TOF–MS/MALDI-TOF-TOF–MS) analysis. Peptide mass fingerprinting data were analysed using the MASCOT server. Peptides with a rank of 1 in the MASCOT search were considered significant and used for the combined peptide score.

### Protein–protein interaction analysis

To investigate the contribution of the identified differentially abundant proteins to the virulence of *M. hyopneumoniae*, we summarised the putative virulence factors reported in previously published papers [3, 21]. The STRING database was used to generate protein–protein interactions between known virulence factors and the novel differentially abundant proteins identified in this study. Only interactions with a confidence score of at least 0.4 were considered for analysis. The protein–protein interaction network was visualised using Cytoscape (3.5.1).

### Core genome analysis

The gene profile (content) of a pan-genome, defined as the entire genomic repertoire of a given species, can be divided into core (shared by all genomes), dispensable, and strain-/isolate-specific genes [32, 33]. The available genomes of nine different *M. hyopneumoniae* strains were downloaded from the NCBI website for core genome analysis [32]. The accession numbers are as follows: strain J, AE017243; strain 168, CP002274; strain 168L, CP003131; strain 232, AE017332; strain 7422, PRJNA47327; strain 7448, AE017244; strain 11, MWWN00000000; strain KM014, CP022714; strain TB1, Scaffold. All genome data are available from NCBI FTP. A pan-genome computation was performed using PGAP v1.2.1, which performs the analysis according to the Heaps low pan-genome models for these genomes. After the input files were built using the Converter_NCBINewFormatData.pl script within PGAP, the PGAP.pl script of PGAP was executed using the Gene Family (GF) method to build a pan-genome profile [34]. Graphs were drawn using an in-house R script.

### Preparation of polyclonal antibody recognising recombinant FBA (rFBA)

The full-length *fba* gene (MHP168_014) was cloned into the pET-28a(+) vector by site-directed mutation and overlap extension (SOE-PCR) due to the existence of rare codons, and rFBA was produced in *Escherichia coli* BL21 (DE3) and purified by Ni-chelating chromatography as described in a previous study [19]. A polyclonal antibody was raised against rFBA by subcutaneously immunising 1-month-old New Zealand white rabbits. Each rabbit was immunised three times with 1 mg of rFBA emulsified in Freund’s adjuvant (Sigma, USA) at 2-week intervals. Sera were collected at 1 week after the third immunisation.

### Western blot validation of comparative proteomics analysis

Equal amounts (40 μg) of each protein sample were separated on a 12% SDS-PAGE gel, and proteins were electrophoretically transferred onto polyvinylidene fluoride (PVDF) membranes (Millipore, Germany) and developed with Ponceau-S as the loading control. After blocking with TBST buffer comprising 20 mM TRIS–HCl (pH 7.6), 150 mM NaCl and 0.1% Tween-20 containing 5% skimmed milk, membranes were probed with anti-rFBA antibody (1:2000 dilution). Horseradish peroxidase (HRP)-conjugated secondary antibody (1:10 000 dilution) was used for final identification. ImageJ software was used to calculate the optical density (OD) of the corresponding bands. The OD of FBA from different samples was normalised to that of the Ponceau-S-stained membrane. The abundance of FBA in *M. hyopneumoniae* strain 168L is expressed as the percentage of that in *M. hyopneumoniae* strain 168. Three replicates were subjected to statistical analysis by SPSS 20.0.

### Detection of surface-exposed FBA by flow cytometry

To investigate whether FBA is present on the surface of *M. hyopneumoniae* strain 168, and to probe FBA surface content differences between 168 and 168L, flow cytometry was performed. In brief, *M. hyopneumoniae* strains 168 and 168L (each 1 × 10^8^ CCU/mL) were incubated with anti-rFBA serum at a 1:100 dilution (1:100 diluted preimmune serum was used as a negative control). *M. hyopneumoniae* cells were then stained with fluorescein isothiocyanate (FITC)-conjugated anti-IgG and the fluorescence intensity was measured using a BD Accuri C6 flow cytometer as described previously [35]. The mean fluorescence intensity (MFI) of *M. hyopneumoniae* incubated with anti-rFBA serum is expressed as the fold-change relative to the corresponding strain incubated with preimmune serum. The assay was performed in triplicate, and data were analysed using Student’s *t* tests in SPSS 20.0. For all tests, *p *≤ 0.05 was considered statistically significant.

### Adherence of rFBA to swine tracheal epithelial cells (STEC)

To investigate the ability of rFBA to promote adherence to STEC, indirect immunofluorescence assays were performed. STEC were grown to confluence in 24-well plates with RPMI-1640 medium (Thermo Fisher Scientific, USA) supplemented with 10% (v/v) fetal bovine serum (Gibco, USA). After incubation with 100 μg of purified rFBA, the cells were washed three times with phosphate-buffered saline (PBS) and incubated with anti-rFBA antibody at a 1:1000 dilution, then with tetraethyl rhodamine isothiocyanate (TRITC)-tagged anti-IgG (Proteintech, 1:500 dilution). Finally, cell nuclei were stained with 6-diamidino-2-phenylindole (DAPI). Fluorescence was detected using a fluorescence microscope (Zeiss, Germany). BSA was used instead of rFBA as a negative control [36].

### Inhibition of adherence using antibody recognising rFBA

*Mycoplasma hyopneumoniae* cells (1 × 10^7^ CCU/mL) were washed three times with PBS and pre-incubated with polyclonal antibody raised against rFBA or preimmune sera (1:20 dilution) at 37 °C for 30 min. Bacteria suspended in RPMI-1640 medium were added to 24-well cell plates containing confluent STEC, and plates were centrifuged at 800 × *g* for 10 min and incubated at 4 °C for 2 h. Bacteria counting including bacterial genome extraction and real-time PCR was performed according to a previous method [19, 37]. The assay was performed in triplicate, and data were analysed using Student’s *t* tests with SPSS 20.0 (*p* ≤ 0.05 was considered statistically significant).

### Far-Western blot analysis of rFBA with fibronectin

To explore whether *M. hyopneumoniae* FBA could bind to fibronectin, the Far-Western blot (Far-WB) protein–protein interaction method was performed. A 20 μg sample of rFBA was separated by SDS-PAGE and transferred to a PVDF membrane [36]. After blocking with 5% (w/v) skimmed milk, the membrane was incubated with 5 µg/mL fibronectin (Sigma), followed by incubation with rabbit anti-fibronectin antibody (Boster; 1 µg/mL) as the primary antibody, and horseradish peroxidase (HRP)-conjugated goat anti-rabbit IgG (Boster; 1:5000 dilution) as the secondary antibody. Finally, the membrane was developed with Electro-Chemi-Luminescence (ECL) substrate using a ChemiDoc XRS+ system (Bio-Rad). BSA was used instead of rFBA as a negative control, and polyclonal antibody against rFBA was used as a positive control.

### Surface plasmon resonance analysis

The interaction dynamics of rFBA and fibronectin were further investigated in real time by surface plasmon resonance (SPR) using a BIAcore X100 Plus instrument (GE Healthcare). Fibronectin was diluted to 10 µg/mL in 10 mM sodium acetate (pH 4.0) and covalently linked to the carboxymethylated dextran matrix of a CM5 sensor chip as the ligand using an amine coupling kit (Biacore AB). Immobilisation of soluble fibronectin generated resonance units (RU) of 2868. Binding kinetics were measured with increasing concentrations (0–100 μg/mL) of the analyte (rFBA) in running buffer (HBS-EP) consisting of 10 mM HEPES, 150 mM NaCl, 3 mM EDTA, and 0.05% (v/v) surfactant P20 (Biacore AB) at a flow rate of 30 μL/min for 180 s over immobilised fibronectin at 20 °C. The dissociation phase was monitored for 1000 s by allowing the buffer to flow over the chip. Association kinetics were analysed manually using Biacore X100 Control Software [11].

## Results

### Clinical observation and lung lesion scoring

**Figure 1:**
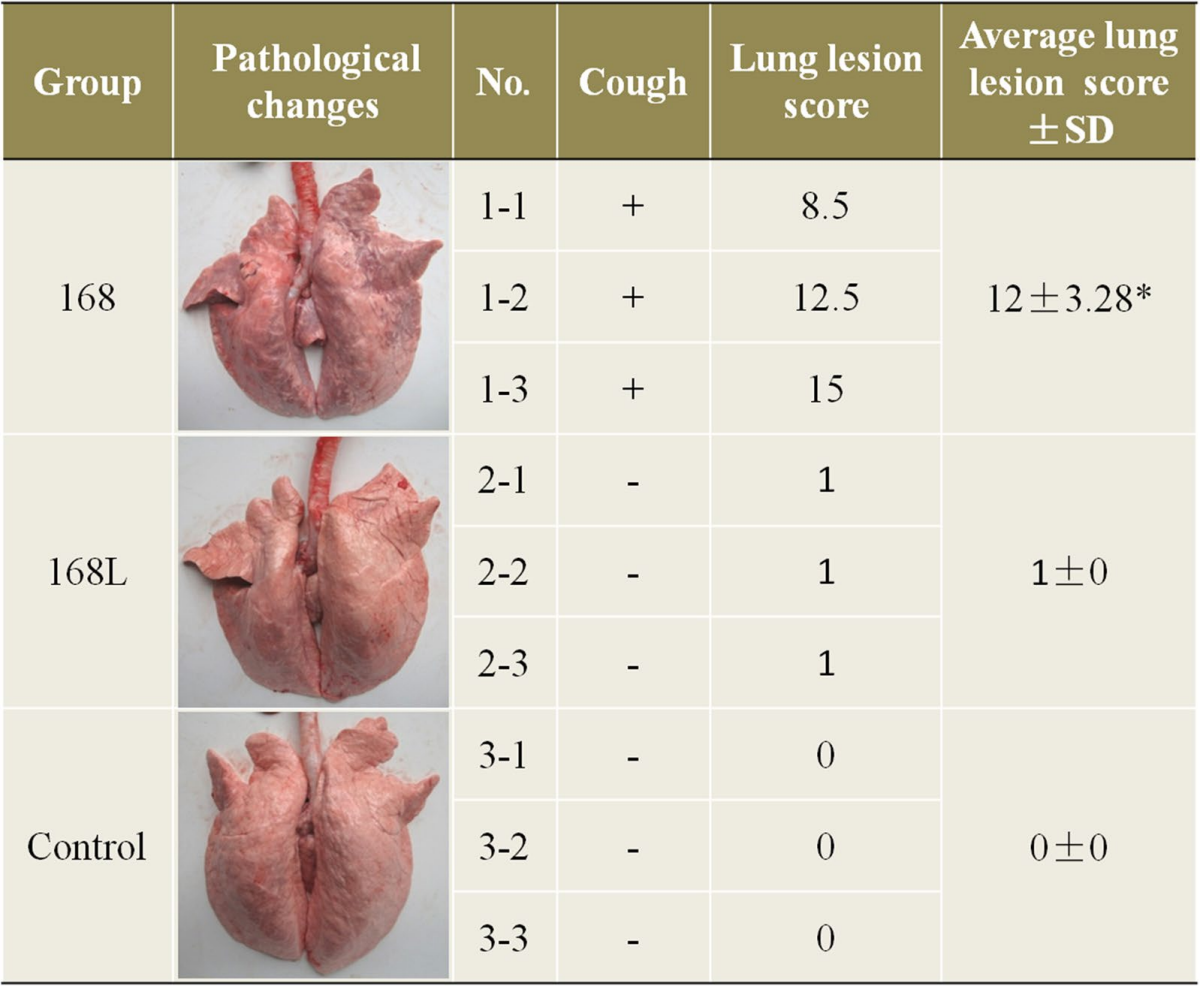
**Evaluation of virulence in** ***Mycoplasma hyopneumoniae*** **strains 168 and 168L.** After treatment, piglets challenged with strain 168 showed symptoms of coughing, but no clinical signs of pneumonia were observed in the 168L or control groups. All pigs were alive during the entire experimental period. Lung lesion scores were subjected to statistical analysis after slaughter. The main lung lesion of the strain 168 challenged group is the pulmonary consolidation in the cranial and middle lobes of the lungs, and the lesions have obvious boundaries with the non-lesioned areas. Significant differences were observed between groups challenged with *M. hyopneumoniae* strain 168 and 168L (*p* < 0.05).

At 14 days after treatment, pigs from the group challenged with *M. hyopneumoniae* strain 168 began to cough, while no clinical signs of pneumonia were observed from the group challenged with *M. hyopneumoniae* strain 168L or the control group. All pigs were alive during the entire experimental period. After euthanising, each of the seven pulmonary lobes was scored for severity of mycoplasmal lesions ranging from 0 to 4 (0 = absence of lobular pneumonia; 1 = 1–25% lesions; 2 = 26–50% lesions; 3 = 51–75% lesions; 4 = 76–100% lesions). The results show that lung lesion levels indicated by the sum of seven pulmonary lobes in the group challenged with *M. hyopneumoniae* strain 168 (average lung lesion score 12) were significantly higher than those in the group challenged with strain 168L (average lung lesion score 1) (Figure 1). The main lesion of the strain 168 challenged group is the pulmonary consolidation in the cranial and middle lobes of lungs, and the lesions have obvious boundaries with the non-lesioned areas. Thus, *M. hyopneumoniae* strain 168 was more virulent than strain 168L.

### Identification of differentially abundant proteins by comparative proteomic analysis

**Figure 2:**
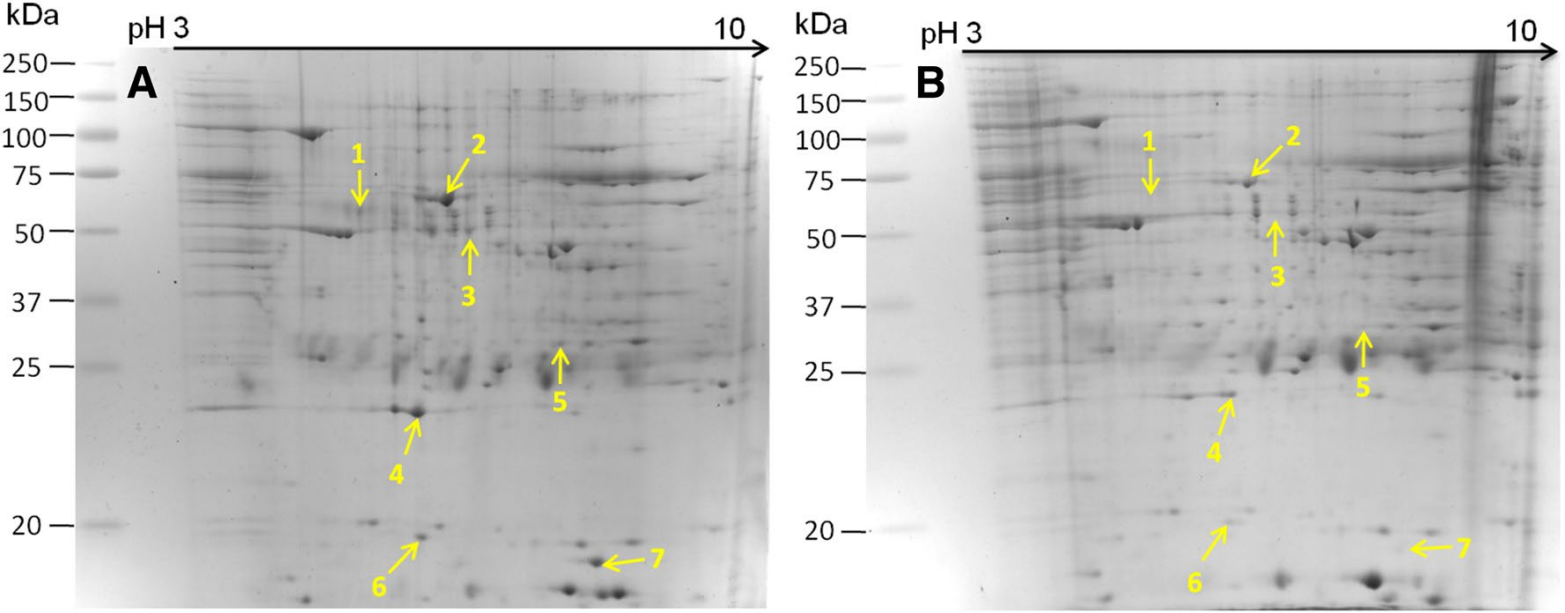
**Identification of differentially abundant proteins by two-dimensional electrophoresis (2-DE). A** Bacterial proteins from *M. hyopneumoniae* strain 168 cultured in KM2 medium. **B** Bacterial proteins from *M. hyopneumoniae* strain 168L cultured in KM2 medium. Yellow arrows on gel images indicate the seven protein spots listed in Table 1 increased in abundance by ≥ 1.5-fold in *M. hyopneumoniae* strain 168.

Proteins ≥ 1.5-fold more abundant in the *M. hyopneumoniae* strain 168 were considered differentially abundant and subjected to MALDI-TOF–MS/MALDI-TOF-TOF–MS analysis. Seven differentially abundant proteins were successfully identified; the molecular chaperone DnaK, elongation factor Tu (EF-Tu), glyceraldehyde 3-phosphate dehydrogenase (GAPDH), adenine phosphoribosyltransferase (Apt), lactate dehydrogenase (LDH), heat shock protein GrpE, and FBA. DnaK, GAPDH and FBA were not detected in lysates of strain 168L by 2-DE analysis, indicating that they were expressed at very low levels in the attenuated strain. However, FBA showed relatively higher expression in *M. hyopneumoniae* 168 based on 2D gels, and received a higher score in MS analysis (Figure 2 and Table 1).

**Table 1:** **The proteins with significant changes in abundance**

Spot no.	Protein description	Fold change	Mascot score^a^	Theoretical pI/Mw	Sequence coverage (%)	Accession
1	Molecular chaperone DnaK (DnaK)	+	179	5.17/68281.56	6	ADQ90292.1
2	Elongation factor Tu (EF-Tu)	1.88	711	5.61/44122.49	27	ADQ90729.1
3	Glyceraldehyde 3-phosphate dehydrogenase (GAPDH)	+	198	6.67/36873.02	11	ADQ90256.1
4	Adenine phosphoribosyltransferase (Apt)	1.94	216	5.34/18849.21	22	ADQ90402.1
5	l-Lactate dehydrogenase (LDH)	1.72	442	8.29/34237.25	20	ADQ90382.1
6	Heat shock protein (GrpE)	1.97	87	5.47/28854.10	6	ADQ90239.1
7	Fructose-bisphosphate aldolase (FBA)	+	342	9.05/43780.88	20	ADQ90241.1

^a^Mascot computes a score based on the probability that the peptides from a sample match those in the selected protein database. The more peptides Mascot identifies from a particular protein, the higher the Mascot score for that protein.

“+” Means those proteins that only observed in the 2-DE map of *M. hyopneumoniae* strain 168, which means its expression level in strain 168L is very low.

### Network analysis of novel differentially abundant proteins and known putative virulence factors

**Figure 3:**
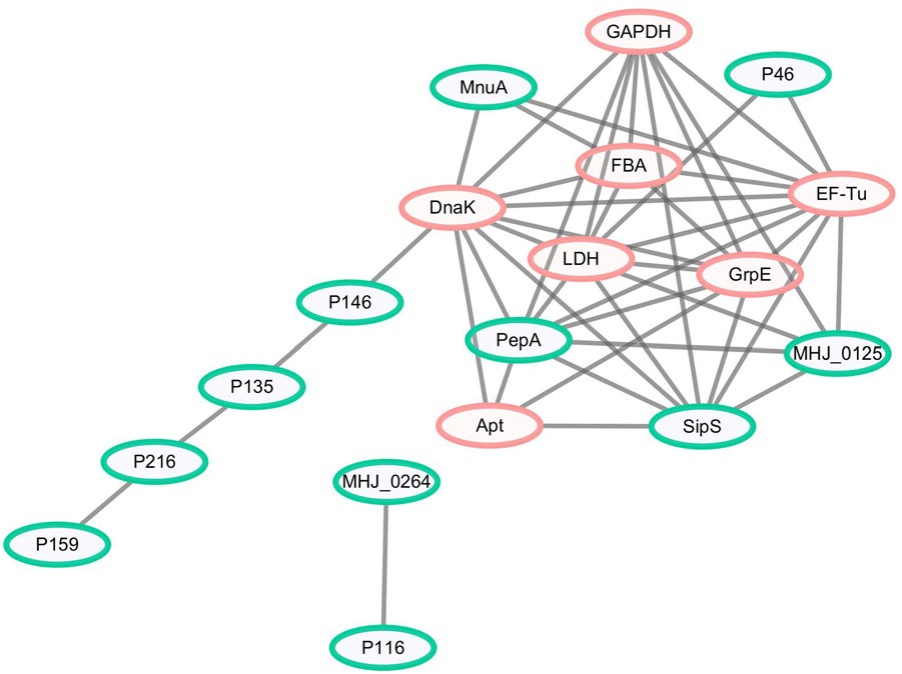
**Interaction networks of the identified differentially abundant proteins and known putative virulence factors.** Protein–protein interactions of differentially abundant proteins with a confidence score ≥ 0.4 are shown. Green nodes represent known putative virulence factors collected from published literature, and orange nodes represent differentially abundant proteins in *M. hyopneumoniae* strain 168 compared with strain 168L. Grey lines represent interactions between two nodes.

A protein–protein interaction network was constructed to explore the possible contributions of the seven differentially abundant proteins to the virulence *of M. hyopneumoniae*. The results revealed a total of 45 direct physical interactions among the 18 nodes (Figure 3 and Additional file 1). Amongst these, 16 interactions had a score > 0.70 (i.e., high confidence), implicating all seven proteins in the interaction network of known virulence factors. The novel differentially abundant proteins (orange nodes) are strongly linked to each other as well as to known virulence factors (green nodes), connected to previously reported putative virulence factors, and form important hub proteins. These results indicate that these seven proteins may play a role in virulence.

### Core genes encoding proteins are involved in *M. hyopneumoniae* virulence

**Figure 4:**
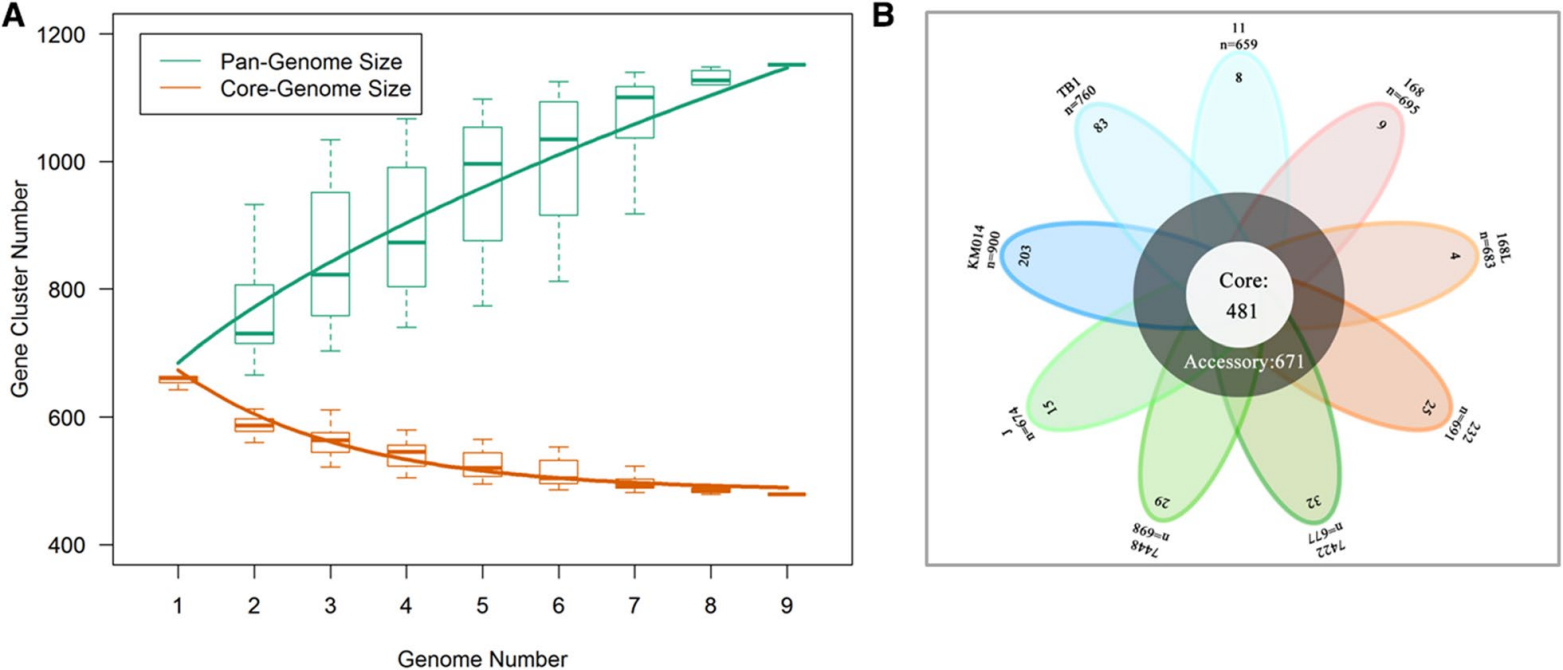
**Predicted size of the pan-genome of** ***M. hyopneumoniae***. **A** Comparative overview of the pan-genome and core genome of *M. hyopneumoniae*. The *M. hyopneumoniae* pan-genome is shown in green (1152 genes), while the core genome is shown in orange (481 genes). Each plot point represents the mean value for gene clusters in the respective number of genomes, and curves represent power law fitting of the data. **B** Flower-plot schematic diagram of all nine *M. hyopneumoniae* strains for which genome data are available, showing the core genome size (flower centre) and the number of unique genes for each strain (flower petals). Numbers below the name of each strain indicate the total number of genes.

Proteome-wide analysis revealed that most of the novel virulence-associated proteins were pivotal enzymes involved in bacterial growth and metabolism. This encouraged us to explore the commonality of virulence-associated factors from a systematic stand point. A pan-genomic analysis with nine available *M. hyopneumoniae* genomes was therefore performed, resulting in 1152 shared genes, including 481 core genes and 671 dispensable genes. The size of the pan-genome increased when a greater number of sequenced genomes are included, but the core genome decreased in size (Figure 4A). The predicted core genome and pan-genome of *M. hyopneumoniae* is shown in the form of a flower-plot schematic diagram (Figure 4B). Unexpectedly, six of the seven novel differentially abundant proteins were encoded by core genes. Only Apt was absent from the genome of *M. hyopneumoniae* strain 11, for which only a draft genome sequence is available (Additional file 2). This indicates that in addition to peripheral or unique genes, core genes can also play an important role in *M. hyopneumoniae* virulence.

### Western blot validation of the results of comparative proteomics analysis

**Figure 5:**
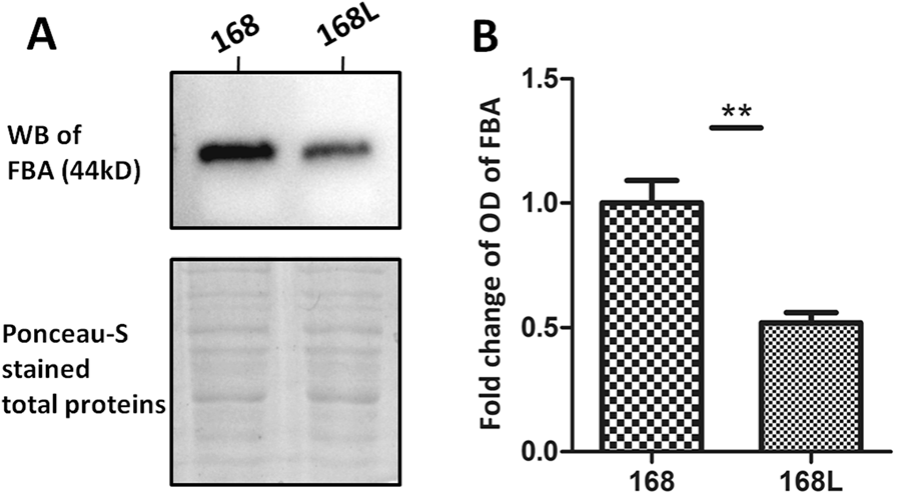
**Western blot analysis of comparative proteomics data. A** The left lane was loaded with bacterial proteins from *M. hyopneumoniae* strain 168. The right lane was loaded with bacterial proteins from strain 168L. The differentially abundant protein fructose-1,6-bisphosphate aldolase (FBA; 44 kDa) was analysed using the corresponding antibodies. Protein bands were visualised using Electro-Chemi-Luminescence (ECL) substrate. **A** Ponceau-S stained membrane was used as the loading control. **B** Image J software was used to calculate the optical density of the corresponding bands in the blots. The optical density of the corresponding bands was normalized to the total proteins of Ponceau-S staining of the same membrane. The level of abundance of FBA in *M. hyopneumoniae* strain 168L is expressed as the percentage of that in *M. hyopneumoniae* strain 168. The asterisk above the charts stands for statistically significant differences.

FBA was selected for validation of the comparative proteomics analysis and for further studies, because it showed the most fold change of all seven differentially abundant proteins, it received a higher score in the MS analysis, and it is encoded by a core gene. The results of Western blotting show that FBA expression was increased significantly in *M. hyopneumoniae* strain 168 compared with that in strain 168L (Figure 5). Thus, upregulation of FBA was shown to be consistent using both approaches.

### Flow cytometry reveals surface localisation of FBA in *M. hyopneumoniae* 168

**Figure 6:**
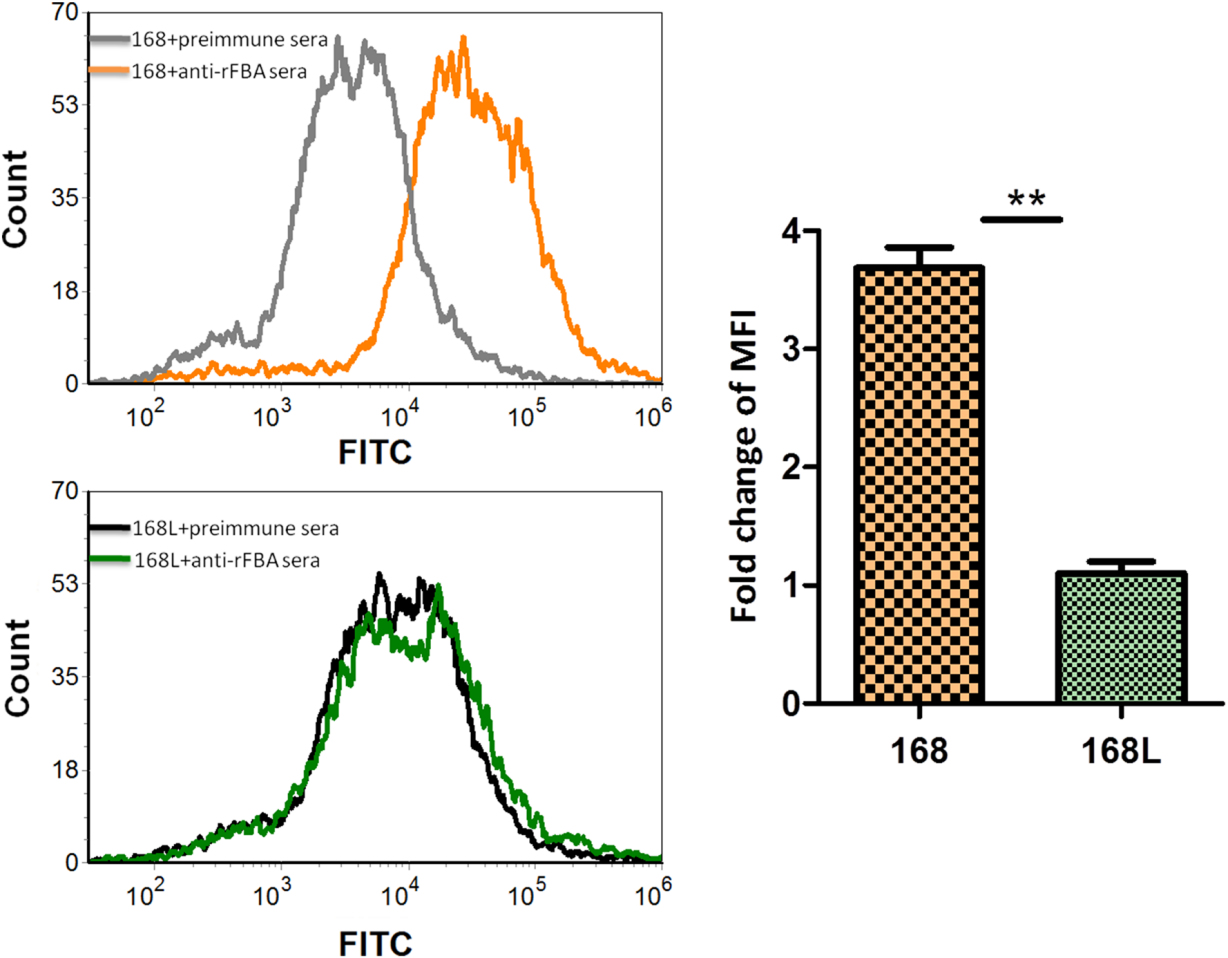
**Detection of FBA on the surface of** ***M. hyopneumoniae*** **by flow cytometry.** Negative control, *M. hyopneumoniae* strain 168 and 168L treated with preimmune serum; *M. hyopneumoniae* strain 168 and 168L: bacteria treated with anti-rFBA serum. The level of mean fluorescence intensity (MFI) of *M. hyopneumoniae* incubated with anti-rFBA sera is expressed as the percentage of the corresponding strain incubated with preimmune sera. The asterisks above the charts stand for statistically significant differences.

There was no significant difference in mean fluorescence intensity (MFI) between *M. hyopneumoniae* strain 168L treated with anti-rFBA serum and strain 168L incubated with preimmune serum, whereas the MFI of strain 168 treated with anti-rFBA serum was 3.7-fold higher than that of strain 168 treated with preimmune serum (Figure 6). Flow cytometry results demonstrate that outer membrane-localised FBA was surface-accessible to FBA-specific antibody in strain 168, FBA antigen was present on the bacterial cell surface of *M. hyopneumoniae* strain 168. In addition, the fold-change in MFI of strain 168 incubated with anti-rFBA compared with preimmune serum was significantly higher than that in strain 168L. This difference indicates that the surface abundance of FBA is significantly higher in strain 168 than in strain 168L.

### Indirect immunofluorescence reveals adherence of rFBA to STEC

**Figure 7:**
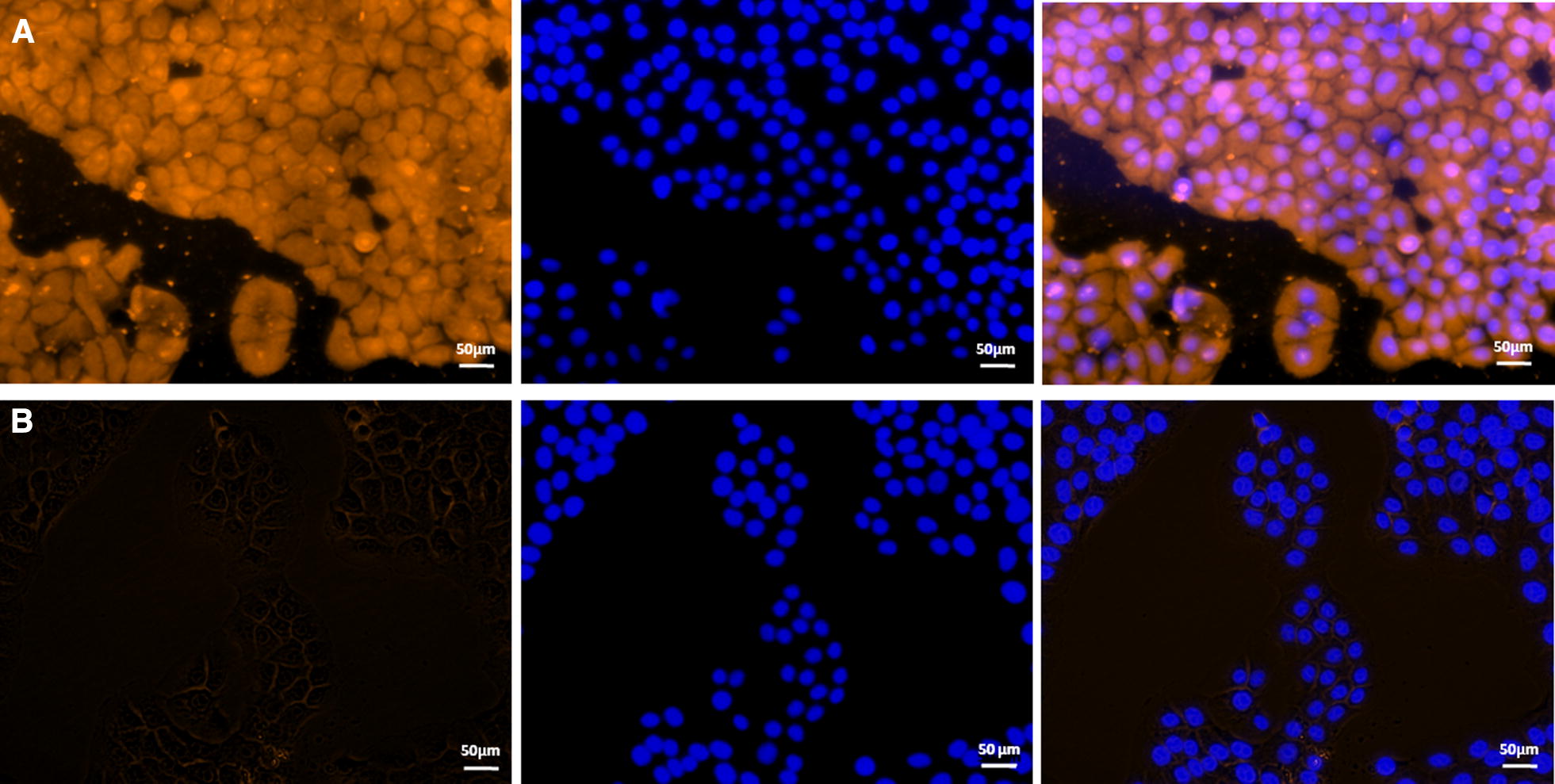
**Role of rFBA in adhesion of** ***M. hyopneumoniae*** **to swine tracheal epithelial cells (STEC).** Blue indicates STEC nuclei. Orange indicates **A** rFBA adhering to STEC membranes, and **B** BSA (negative control) adhering to STEC membranes. The left panel means protein adhered to the STEC membranes. The protein was labelled with tetraethyl rhodamine isothiocyanate (TRITC). The middle panel means cell nuclei of STEC stained with 6-diamidino-2-phenylindole (DAPI). The right panel means the merge of the left and middle panel. The white line indicates the scale.

To explore the potential mechanism(s) by which surface localisation of FBA affects virulence, we used indirect immunofluorescence to determine whether rFBA could adhere to STEC. The results revealed significant fluorescence on the cell surface of STEC incubated with rFBA (Figure 7A), but no specific fluorescence was observed around DAPI-stained cell nuclei in negative controls (Figure 7B). The results provide direct evidence that rFBA binds specifically to the cell membranes of STEC.

### Confirmation of adherence by antibody inhibition assay

**Figure 8:**
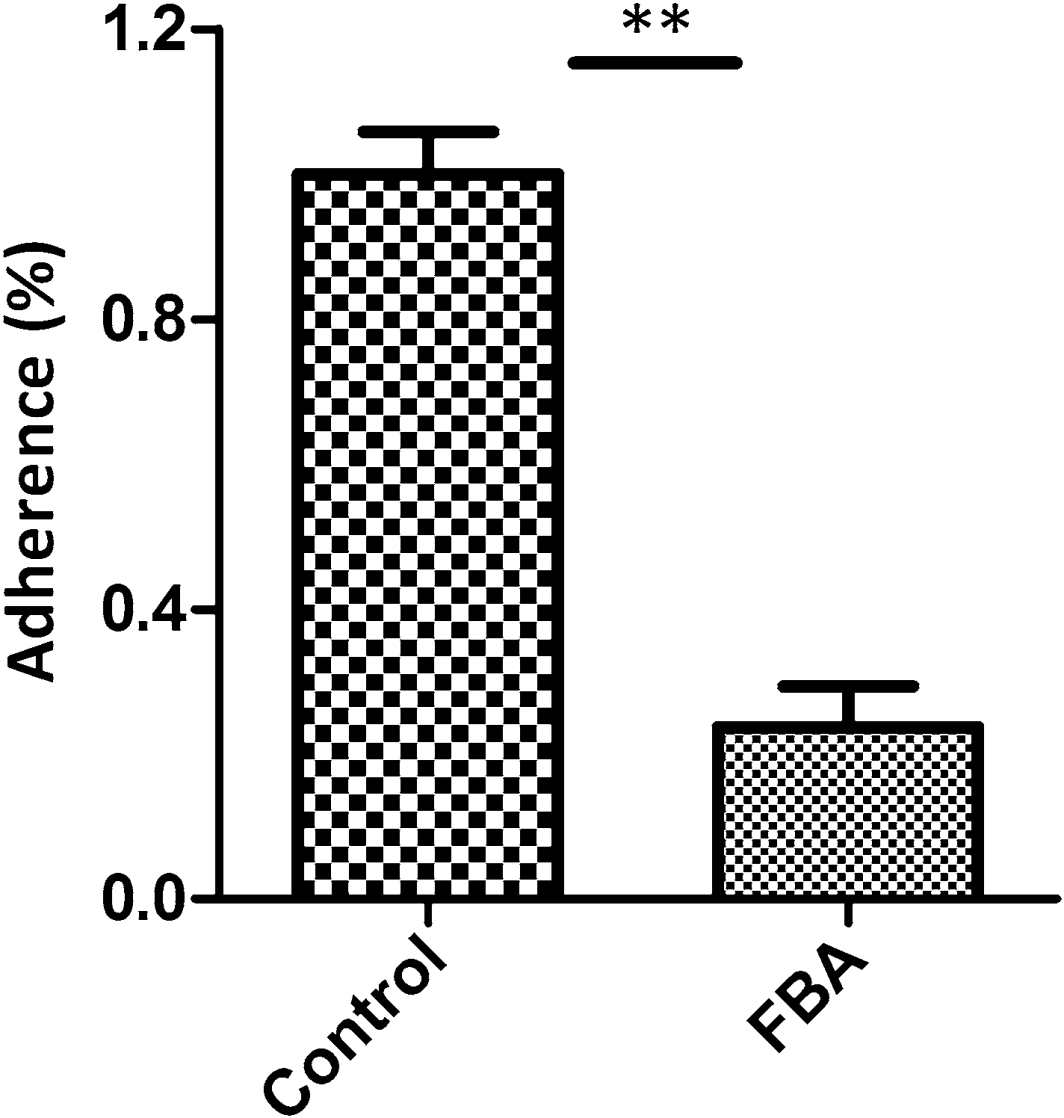
**Inhibition of** ***M. hyopneumoniae*** **adhesion to STEC.** Adhesion rate = (number of bacteria recovered from cells incubated with anti-rFBA sera/number of bacteria recovered in the group incubated with preimmune sera) × 100. Data are expressed as mean ± SD of at least three experiments with samples performed in triplicate. The asterisks above the charts stand for statistically significant differences.

Antibody inhibition assays were performed to further assess the contribution of surface-localised FBA to adhesion in *M. hyopneumoniae* strain 168. A polyclonal antibody against rFBA was found to decrease *M. hyopneumoniae* adherence to STEC relative to treatment with preimmune sera (Figure 8). The level of adherence is expressed as the percentage of *M. hyopneumoniae* adherence without antibody. Incubation with anti-rFBA antibody resulted in a 76% (*p* < 0.05) reduction in the adherence efficiency of *M. hyopneumoniae* to STEC, further confirming that FBA plays an indispensable role in adherence of *M. hyopneumoniae* to host cells.

### rFBA binds specifically to fibronectin

**Figure 9:**
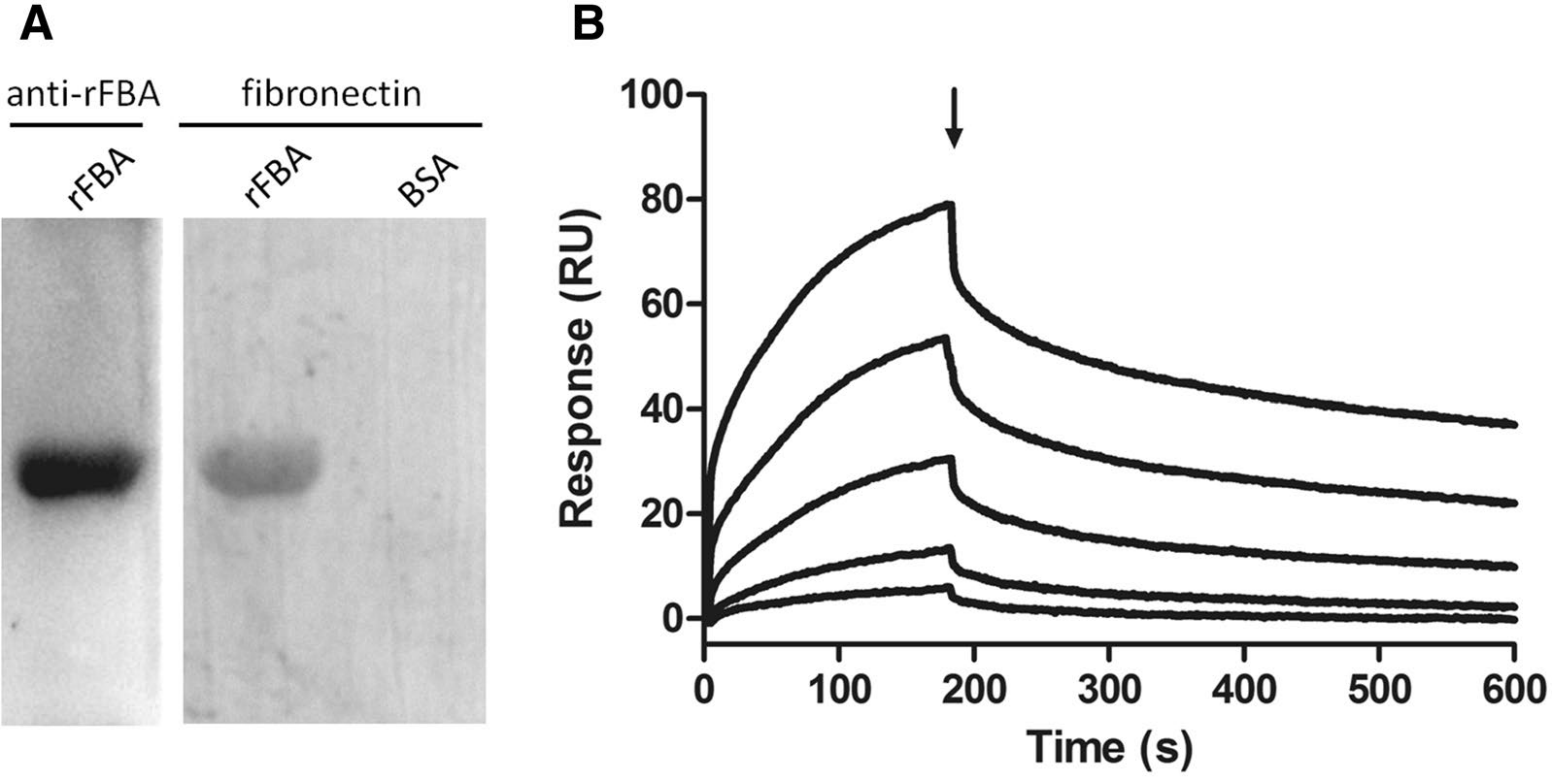
**rFBA and fibronectin interaction analysis by Far-WB and Surface plasmon resonance (SPR) analysis. A** Far-WB analysis of rFBA with fibronectin. The first lane: PVDF membrane with transferred rFBA protein incubated with anti- rFBA antibody as a positive control; the second lane: PVDF membrane with transferred rFBA protein incubated with fibronectin and anti-fibronectin antibody; the third lane: PVDF membrane with transferred BSA (negative control) incubated with fibronectin and anti-fibronectin antibody. Protein bands were visualized using ECL substrate. **B** Sensorgrams depict the binding of immobilised fibronectin to rFBA. Increasing concentrations of rFBA (5, 10, 25, 50 and 100 μg/mL) were injected at a flow rate of 30 μL/min for 180 s over immobilised fibronectin. The arrow indicates the end of the injection period, at which point dissociation of rFBA from fibronectin can be observed. *RU* resonance units.

To investigate the STEC components that interact with FBA, we examined the fibronectin-binding activity of rFBA using Far-WB analysis. The corresponding bands were observed in both reactions of rFBA to anti-FBA antibody (positive control) and to fibronectin, while no specific reaction was observed in the negative control. The analysis indicates that rFBA could specifically bind to fibronectin (Figure 9A). Using surface plasmon resonance (SPR), the real-time interactions between rFBA and fibronectin were further investigated (Figure 9B). The results were consistent with those expected for a specific, moderately strong interaction between proteins of this size, and rFBA was found to bind fibronectin in a dose-dependent and physiologically relevant manner, with *K*_D_ = 468 ± 23 nM and *k*_a_ = 3470 ± 580 M^−1^ s^−1^.

## Discussion

The pan-genome includes the core genome containing genes present in all individuals, the accessory or dispensable genome containing genes present in two or more strains, and unique genes specific to single strains [33]. The expression of special elements encoded in the accessory genome, especially unique genomic elements carried by pathogenic strains, is generally considered to be associated with virulence [38]. For example, *Escherichia coli strains* include 1200 genes in the conserved core genome, but more than 13 000 gene families in the pan-genome. This is in part due to several major virulence factors being located on transmissible genetic elements such as pathogenicity islands, bacteriophages, or plasmids, which are classed as accessory genes [38]. Similarly, Shiga toxin, encoded by *stx*, *is* a bacteriophage element [39], and the adhesion and Shiga virulence factor intimin is encoded by *eae*, which belongs to a pathogenicity island.

Although accessory genes encode many conspicuous determinants of virulence, core genes can also confer appreciable virulence. Indeed, more and more moonlighting proteins are being recognised with roles in bacterial pathogenesis [40]. Moonlighting proteins, exhibiting two or more biochemical functions using a single polypeptide chain [41], are commonly encoded by the core genome, and are often enzymatically active with cytoplasmic roles in glycolysis or other metabolic pathways. However, such proteins are often displayed on the surface of bacteria, where they perform functions unrelated to their cytoplasmic roles that may be associated with virulence, and many interact with a variety of host ligands [42].

In the present study, 2-DE analysis revealed that seven proteins were significantly increased in abundance in the virulent *M. hyopneumoniae* strain. A virulence-associated network constructed using these seven proteins and previously reported putative virulence factors showed that all seven novel differentially abundant proteins are involved in *M. hyopneumoniae* virulence. Since all seven are conserved enzymes, we explored a possible common pattern among these virulence-associated factors from a system and whole-genome perspective. A pan-genomics analysis with nine available *M. hyopneumoniae* genomes was therefore performed. Unexpectedly, six of the seven novel differentially abundant proteins are encoded by core genes, and the only protein potentially encoded by an accessory gene may not be in reality due to incomplete sequencing of strain 11. This indicates that core genes, which are often neglected, can play an important role in *M. hyopneumoniae* virulence.

Of the seven proteins, FBA showed the most fold change in different virulence *M. hyopneumoniae* strains, and this protein is a promising therapeutic and vaccine target in bacteria [43]. FBA is a ubiquitous metabolic enzyme occupying a central position in glycolysis and gluconeogenesis pathways [44]. Two different classes of FBA (Class-I and -II) have been described based on amino acid sequences [45]. Class-I FBA are usually found in higher eukaryotic organisms (animals and plants), while Class-II FBA are commonly found in bacteria, archaea and lower eukaryotes [46].

Despite lacking identifiable secretion signals, FBA are also localised at the bacterial cell surface, where they interact directly with host proteins and exhibit non-glycolytic functions. Remarkably, FBA has been recently reported to play a role in the pathogenesis of several pathogens. FBA is antigenic in humans, and affords significant protection against challenge with virulent *Streptococcus pneumoniae* in mice [47]. *S. pneumoniae* FBA is a cell wall-localised protein, and anti-rFBA antibodies can inhibit *S. pneumoniae* adherence to epithelial cells. Flamingo cadherin has been identified as the host receptor [48]. FBA from *Streptococcus suis*, which is reasonably well conserved among *S. suis* strains, was verified as an immunogenic cell wall protein, suggesting it could be developed as a vaccine candidate [49]. FBA of *Neisseria meningitidis* is localised both in the cytoplasm and the outer membrane, and is required for adhesion to human cells [50] by binding to plasminogen [51]. In *Mycobacterium tuberculosis*, FBA is required for survival during the chronic phase of mouse infection [52]. FBA has also been shown to be essential for replication and virulence in *Toxoplasma gondii* [53], and FBA from *Francisella novicida* is important for bacterial multiplication in macrophages, and plays a regulatory role in pathogenesis [44].

Our flow cytometry analysis indicates that FBA was accessible on the surface of the highly virulent *M. hyopneumoniae* 168 strain, but less so on the attenuated 168L strain. Adherence of viable *M. hyopneumoniae* strain 168 to STEC was inhibited significantly by anti-rFBA antisera, which indicates that FBA is indispensable for adherence to STEC. These results demonstrate that *M. hyopneumoniae* FBA, in addition to its major cytoplasmic, biosynthetic, and metabolic roles, can translocate to the surface and moonlight as an important adhesion factor.

Colonising the epithelial surfaces of the respiratory tract is an important part of the pathogenesis of *M. hyopneumoniae*. Since adherence to host tissues is an important prerequisite for colonisation and subsequent disease development for pathogenic bacteria, adhesins are of crucial importance for *M. hyopneumoniae* infection [54]. Bacterial adhesion factors frequently interact with extracellular matrix (ECM) components, most commonly fibronectin [55, 56], an abundant glycoprotein deposited on cell surfaces [57]. Immunohistochemical staining showed that fibronectin is freely available in the trachea and bronchioles of the porcine lung, especially along the borders of cilia [19], the most common site of colonisation for *M. hyopneumoniae*. The capacity to bind fibronectin is widespread in bacterial pathogens, and among mycoplasmas, the first fibronectin interaction was identified in *M. penetrans* [58], followed by *M. pneumoniae* [59] and *M. hyopneumoniae* [11]. Many fibronectin-binding proteins have been identified, such as the microbial surface components recognising the adhesive matrix molecule (MSCRAMM) family of proteins identified in *Streptococcus* [60] and *Staphylococcus* [61]. The mammalian fibronectin system is widely-used by pathogens as a virulence strategy. In our previous work, blocking fibronectin in STEC decreased *M. hyopneumoniae* adherence to the cell surface. Consistently, fibronectin was verified as one of the host cell receptors for *M. hyopneumoniae* adhesion [19]. The dissociation constant for rFBA and fibronectin determined by SPR was *K*_D_ of 468 nM, signifying a specific and moderately strong interaction that may be physiologically relevant. This is the first report of an interaction between FBA of *M. hyopneumoniae* and host fibronectin contributing to adherence and therefore pathogenicity.

In summary, our research indicates the importance of core genes in the virulence of *M. hyopneumoniae*. Specifically, FBA is abundant on the surface of virulent *M. hyopneumoniae* strains, where it binds strongly to host fibronectin, thereby promoting adherence to STEC. FBA can be surface translocated and may play a role in the adhesion to host cells and colonisation, thereby serving as an important candidate virulence factor of *M. hyopneumoniae*. Thus, we can conclude that core enzymes may be important virulence determinants. To our knowledge, this is the first report describing the moonlighting function of FBA in *M. hyopneumoniae* virulence, and the first to present evidence of the involvement of core genes in virulence in this species. Intrinsic virulence functions represent potential targets for broad-spectrum drugs, and links between intrinsic gene functions and virulence traits are clearly worthy of further study. For organisms with relatively small genomes, multi-functional proteins may be particularly useful for optimising the potential of the genome [50]. However, only nine *M. hyopneumoniae* genomes are currently available, which may limit pan-genome analysis to some extent. Indeed, only partial inhibition of FBA was observed in competitive adhesion inhibition assays.

In conclusion, the core genome encoding proteins located in the cytoplasm and the cell membrane appears to be associated with virulence in *M. hyopneumoniae*. Much remains to be elucidated about how proteins lacking signal motifs are localised on the bacterial cell surface. The roles of core genome encoding proteins in infection and immunity in *M. hyopneumoniae* and other pathogenic organisms are clearly worthy of further investigation.

The combined comparative proteomics and core genomics analyses employed herein successfully identified potential virulence-associated factors in *M. hyopneumoniae*. Among these factors, FBA does not have a pig ortholog, making it a prime candidate for a swine pneumonia vaccine. Our comprehensive analysis revealed seven virulence-associated factors, at least six of which are encoded in the core genome of *M. hyopneumoniae*. The current lack of effective vaccine candidates and the global expansion of sequencing data make this a potentially powerful approach for the identification and development of effective vaccines against *M. hyopneumoniae* and other bacterial pathogens.

## Additional files

**Additional file 1.** **Protein–protein interaction network.** Network analysis of novel differentially abundant proteins and known putative virulence factors of *M. hyopneumoniae* by String database.

**Additional file 2.** **Pan-genomic analysis.** A pan-genomic analysis with nine available *M. hyopneumoniae* genomes.
